# HIV testing trends among gay men in Scotland, UK (1996–2005): implications for HIV testing policies and prevention

**DOI:** 10.1136/sti.2008.033886

**Published:** 2009-03-09

**Authors:** L M Williamson, P Flowers, C Knussen, G J Hart

**Affiliations:** 1 MRC Social and Public Health Sciences Unit, Glasgow, UK; 2 Division of Psychology, School of Life Sciences, Glasgow Caledonian University, Glasgow, UK; 3 Centre for Sexual Health and HIV Research, Research Department of Infection and Population Health, University College London, London, UK

## Abstract

**Objective:**

To examine trends in the HIV testing behaviour of gay men in Scotland over a 10-year period.

**Methods:**

Seven cross-sectional surveys in commercial gay venues in Glasgow and Edinburgh (1996–2005). 9613 men completed anonymous, self-completed questionnaires (70% average response rate).

**Results:**

Among 8305 respondents included in these analyses, HIV testing increased between 1996 and 2005, from 49.7% to 57.8% (p<0.001). The proportion of men who had tested recently (in the calendar year of, or immediately before, the survey) increased from 28.4% in 1996 to 33.2% in 2005, when compared with those who have tested but not recently, and those who have never tested (adjusted odds ratio 1.31, 95% CI 1.13 to 1.52). However, among ever testers, there was no increase in rates of recent testing. Recent testing decreased with age: 31.3% of the under 25, 30.3% of the 25–34, 23.2% of the 35–44 and 21.2% of the over 44 years age groups had tested recently. Among men reporting two or more unprotected anal intercourse partners in the previous year, only 41.4% had tested recently.

**Conclusions:**

HIV testing among gay men in Scotland increased between 1996 and 2005, and corresponds with the Scottish Government policy change to routine, opt-out testing in genitourinary medicine clinics. Testing rates remain low and compare unfavourably with near-universal testing levels elsewhere. The limited change and decline across age groups in recent HIV testing rates suggest few men test repeatedly or regularly. Additional, innovative efforts are required to increase the uptake of regular HIV testing among gay men.

Increases in HIV incidence among men who have sex with men (MSM) have been reported,^1^ and MSM remain the group most at risk of acquiring HIV in the UK.^2^ In Scotland, MSM account for 36% of HIV diagnoses, and current HIV prevalence in this group is 4.3%.^3^ In 2005, community-based surveys of gay men in Scotland found 42% of HIV-positive men were undiagnosed,^4^ compared with 30% of MSM genitourinary medicine (GUM) clinic attenders, but recent estimates suggest less than 10% remain undiagnosed following a clinic attendance.^3^ This has been credited to the introduction of routine, opt-out testing in GUM clinics, whereby all patients should be offered an HIV test regardless of symptoms or risk factors. This was implemented as part of the Scottish sexual health strategy,^5^ which was distinct from the strategy for England and Wales.^6^ This policy is now recommended throughout the UK.^7^

The promotion of regular and frequent HIV antibody testing as a means of identifying, and therefore limiting, the potential onward transmission of infection is a core component of prevention efforts in the USA and the Centers for Disease Control and Prevention recently recommended routine HIV testing should be performed in all healthcare settings.^8^ In the UK, HIV testing has traditionally played a lesser part in prevention efforts but its promotion has been central to recent campaigns (see, for example, http://www.hivcomebacktour.co.uk/). The UK Chief Medical Officer recently advocated extension of this to all healthcare settings,^9^ and the 2008 UK national guidelines for HIV testing are designed to facilitate this.^7^ However, HIV testing rates among gay men in Scotland have traditionally been lower than among men in similar surveys elsewhere in the UK and Europe,^10^ ^11^ and compare unfavourably with the near universal rates reported in the USA and Australia.^12^ ^13^ In this paper, we examine trends in the HIV testing behaviour of gay men in Scotland over a 10-year period, and discuss whether we are doing enough to promote testing in the group most at risk of acquiring HIV in Scotland.

## Methods

We conducted seven cross-sectional surveys between 1996 and 2005 in commercial gay venues in Glasgow and Edinburgh (table 1).^4^ ^14^ ^15^ ^16^ Time and location sampling was used to recruit representative samples. All seven surveys utilised anonymous, self-completed questionnaires and respondents were asked whether they had ever had an HIV test and for the date of their most recent test. Those tested in the year of the survey, or the year immediately before it, were categorised as recent HIV testers (eg, for the 2005 survey, men testing in 2005 or 2004 were categorised as recent testers).

**Table 1:** Survey year, source, location and numbers approached, participating and included in the analyses

Year of survey	Source	Location	Number approached	Number participating (N = 9613)	Response rate (%)	Number included in analyses (N = 8305)*
1996	MRC	Edinburgh/Glasgow	2881	2276	79	2097
1999	MRC	Edinburgh/Glasgow	3202	2498	78	2125
2000	HGS	Edinburgh/Glasgow	1029	803	78	652
2002^a^	MRC	Edinburgh/Glasgow	2796	1734	62	1468
2002^b^	GMH	Edinburgh	456	283	62	262
2003	GMH	Edinburgh	429	275	64	230
2005	MRC	Edinburgh/Glasgow	2642	1744	66	1471

*Those who responded to all of the following questions: age, ever had HIV test, date of last HIV test and numbers of unprotected anal intercourse partners in the previous year. GMH, Gay Men’s Health; HGS, Healthy Gay Scotland; MRC, Medical Research Council.

Ethics approval was granted for the 1996, 1999, 2002a, and 2005 surveys by the University of Glasgow Ethics Committees for Non-clinical Research Involving Human Subjects, and for the 2000 survey by the Psychology Ethics Committee at Glasgow Caledonian University. The 2002b and 2003 surveys were exact replications of the 2000 study design and methodology. Consequently no further ethics approval for these surveys was sought.

The data were analysed by χ^2^ tests and logistic regression. The analysis was conducted using SPSS 16.0 for Mac.

## Results

Questionnaires were obtained from 9613 men with an average response rate of 70% (table 1). Men responding to the question on HIV testing, and who also provided information on age, date of last HIV test and numbers of unprotected anal intercourse (UAI) partners in the previous year, are included in the analysis (N  =  8305). The age breakdown of each survey is shown in table 2.

**Table 2:** Age of respondents by year of survey (N  =  8305)

	Year of survey						
	1996	1999	2000	2002a	2002b	2003	2005
	N = 2097	N = 2125	N = 652	N = 1468	N = 262	N = 230	N = 1471
	N (%)	n (%)	n (%)	n (%)	n (%)	n (%)	n (%)
Age group, years							
<25	475 (22.7)	479 (22.5)	173 (26.5)	438 (29.8)	82 (31.3)	43 (18.7)	400 (27.2)
25–34	1010 (48.2)	942 (44.3)	263 (40.3)	567 (38.6)	96 (36.6)	77 (33.5)	488 (33.2)
35–44	455 (21.7)	504 (23.7)	161 (24.7)	366 (24.9)	63 (24.0)	77 (33.5)	407 (27.7)
>44	157 (7.5)	200 (9.4)	55 (8.4)	97 (6.6)	21 (8.0)	33 (14.3)	176 (12.0)

Overall, 4370 respondents (52.6%) reported that they had had an HIV test. The proportion reporting they had ever had an HIV test increased between 1996 and 2005, from 49.7% to 57.8% (p<0.001). Table 3 shows the proportions who reported having a recent test (in the calendar year of, or immediately before, the survey), having ever, but not recently, tested (within >1–5 and 6+ years ago) and having never tested by age group and year of survey. Although the proportion who reported recent testing increased from 28.4% in 1996 to 33.2% in 2005 (reaching their highest level of 40.4% in 2003), the proportion tested over 1–5 years ago varied slightly over the seven surveys but remained at 15% in 1996 and 2005, and the proportion tested more than 6 years ago increased from 6.2% in 1996 to 9.6% in 2005. The odds ratios for recent testing (compared with those who have tested but not recently and those who have never tested), adjusted for age, are shown in table 3. Compared with 1996, the odds ratios associated with recent testing for the 1999 and 2002a surveys were somewhat lower (p = 0.003 and p = 0.038, respectively), whereas the odds ratio for the 2000 survey did not differ significantly from that associated with 1996. However, the odds ratios for the 2002b, 2003 and 2005 surveys were all significantly higher (all p<0.01), indicating that recent testing rates in these years were higher than those recorded in 1996.

**Table 3:** HIV testing by year of survey and age of respondents (N  =  8305)

	Year of survey						
	1996	1999	2000	2002a	2002b	2003	2005
	N = 2097	N = 2125	N = 652	N = 1468	N = 262	N = 230	N = 1471
	n (%)	n (%)	n (%)	n (%)	n (%)	n (%)	n (%)
Age group							
<25 Years							
Recent HIV test*	161 (33.9)	127 (26.5)	57 (32.9)	118 (26.9)	33 (40.2)	22 (51.1)	137 (34.2)
Last tested >1–5 years ago	40 (8.4)	49 (10.2)	12 (6.9)	50 (11.4)	4 (4.9)	3 (7.0)	31 (7.8)
Last tested 6+ years ago	4 (0.8)	3 (0.6)	0 (0)	0 (0)	0 (0)	0 (0)	6 (1.5)
Never had HIV test	270 (56.8)	300 (62.6)	104 (60.1)	270 (61.6)	45 (54.9)	18 (41.9)	226 (56.5)
25–34 Years							
Recent HIV test*	300 (29.7)	245 (26.0)	71 (27.0)	155 (27.3)	37 (38.5)	32 (41.6)	202 (41.4)
Last tested >1–5 years ago	179 (17.7)	194 (20.6)	66 (25.1)	131 (23.1)	20 (20.8)	17 (22.1)	78 (16.0)
Last tested 6+ years ago	68 (6.7)	62 (6.6)	17 (6.5)	43 (7.6)	7 (7.3)	7 (9.1)	27 (5.5)
Never had HIV test	463 (45.8)	441 (46.8)	109 (41.4)	238 (42.0)	32 (33.3)	21 (27.3)	181 (37.1)
35–44 Years							
Recent HIV test*	102 (22.4)	102 (20.2)	38 (23.6)	76 (20.8)	18 (28.6)	30 (39.0)	106 (26.0)
Last tested >1–5 years ago	74 (16.3)	109 (21.6)	29 (18.0)	84 (23.0)	15 (23.8)	16 (20.8)	86 (21.1)
Last tested 6+ years ago	47 (10.3)	60 (11.9)	24 (14.9)	58 (15.8)	11 (17.5)	16 (20.8)	74 (18.2)
Never had HIV test	232 (51.0)	233 (46.2)	70 (43.5)	148 (40.4)	19 (30.2)	15 (19.5)	141 (34.6)
>44 Years							
Recent HIV test*	32 (20.4)	37 (18.5)	9 (16.4)	20 (20.6)	7 (33.3)	9 (27.3)	43 (24.4)
Last tested >1–5 years ago	25 (15.9)	30 (15.0)	5 (9.1)	15 (15.5)	2 (9.5)	8 (24.2)	26 (14.8)
Last tested 6+ years ago	10 (6.4)	31 (15.5)	13 (23.6)	16 (16.5)	4 (19.0)	4 (12.1)	34 (19.3)
Never had HIV test	90 (57.3)	102 (51.0)	28 (50.9)	46 (47.4)	8 (38.1)	12 (36.4)	73 (41.5)
Total							
Recent HIV test*	595 (28.4)	511 (24.0)	175 (26.8)	369 (25.1)	95 (36.3)	93 (40.4)	488 (33.2)
Last tested >1–5 years ago	318 (15.2)	382 (18.0)	112 (17.2)	280 (19.1)	41 (15.6)	44 (19.1)	221 (15.0)
Last tested 6+ years ago	129 (6.2)	156 (7.3)	54 (8.3)	117 (8.0)	22 (8.4)	27 (11.7)	141 (9.6)
Never had HIV test	1055 (50.3)	1076 (50.6)	311 (47.7)	702 (47.8)	104 (39.7)	66 (28.7)	621 (42.2)
AOR† of having recently had HIV test (95% CI)	1	0.81 (0.71 to 0.93)	0.94 (0.77 to 1.15)	0.85 (0.73 to 0.99)	1.45 (1.11 to 1.91)	1.87 (1.41 to 2.48)	1.31 (1.13 to 1.52)

*Recent HIV testing includes all men tested in the calendar year of the survey and the calendar year before the survey (eg, for the 2005 survey, all men tested in 2004 and 2005 are included in the recent testers category). †Adjusted odds ratio (AOR) of having had a recent HIV test by year of survey, adjusted for age.

However, recency of testing among those who reported having had an HIV test at some point in their lives varied but there was no real increase over time, falling from 57.1% (595/1042) in 1996 to 48.2% (369/766) in 2002a and rising to 57.5% (488/850) in 2005. The adjusted odds ratios (AOR) for recent testing compared with those who had ever, but not recently, tested (excluding those who have never tested) were also calculated. Compared with 1996, the odds ratios were significantly different (lower) in the 1999 (AOR 0.79, 95% CI 0.62 to 0.89) and 2002a (AOR 0.69, 95% CI 0.57 to 0.84) surveys. However, the odds ratios were not significantly different in the 2002b (AOR 1.16, 95% CI 0.81 to 1.65), 2003 (AOR 1.17, 95% CI 0.83 to 1.65) or 2005 (AOR 1.10, 95% CI 0.91 to 1.33) surveys. This indicates that among men who had had an HIV test, there was no increase in rates of recent testing between 1996 and 2005.

Although the proportion of men who had never had an HIV test decreased with age between the under 25, 25–34 and 35–44 years age groups, the proportion tested recently also decreased with age. On average, 31.3% of the under 25, 30.3% of the 25–34, 23.2% of the 35–44 and 21.2% of the over 44 years age groups had tested recently. Table 3 shows the pattern of change over time for each age group. Among the under 25 years age group, recent testing fell from 33.9% in 1996 to 26.9% in 2002a and rose to 34.2% in 2005 (with a high of 51.1% in the Edinburgh-only 2003 survey). Among the 25–34 years age group, recent testing increased from 29.7% in 1996 to 41.4% in 2005. In the 35–44 years age group, recent testing rose from 22.4% in 1996 to 39.0% in 2003 and fell to 26.0% in 2005. In the over 44 years age group, the proportions tested recently varied over time, rising from 20.4% in 1996 to 24.4% in 2005.

Aggregate rates of recent HIV testing were consistently higher among men who reported two or more UAI partners in the previous year than among men who reported none or one partner (AOR 1.93, 95% CI 1.68 to 2.23; table 4). Overall, 41.4% of men reporting UAI with two or more partners had tested recently. Among men who reported UAI with two or more partners, recent testing was lowest in the 35–44 year age group, with only 34.2% having had a recent test.

**Table 4:** Recent HIV testing by reported number of UAI partners in the previous year and age of respondents

	Age group				
	<25 Years	25–34 Years	35–44 Years	45+ Years	Total
	N = 2090	N = 3443	N = 2033	N = 739	N = 8305
	n (%)	n (%)	n (%)	n (%)	n (%)
No of UAI partners in previous year					
0/1 Partner					
Had recent HIV test*	539 (29.8)	870 (28.4)	407 (22.1)	131 (19.5)	1947 (26.3)
Last tested >1–5 years ago	162 (9.0)	616 (20.1)	377 (20.4)	98 (14.6)	1253 (17.0)
Last tested 6+ years ago	13 (0.7)	212 (6.9)	263 (14.3)	106 (15.8)	594 (8.0)
Never had HIV test	1093 (60.5)	1369 (44.6)	796 (43.2)	337 (50.1)	3595 (48.7)
2+ Partners					
Had recent HIV test*	116 (41.0)	172 (45.7)	65 (34.2)	26 (38.8)	379 (41.4)
Last tested >1–5 years ago	27 (9.5)	69 (18.3)	36 (18.9)	13 (19.4)	145 (15.8)
Last tested 6+ years ago	0 (0)	19 (5.1)	27 (14.2)	6 (9.0)	52 (5.7)
Never had HIV test	140 (49.5)	116 (30.9)	62 (32.6)	22 (32.8)	340 (37.1)

*Recent HIV testing includes all men tested in the calendar year of the survey and the calendar year before the survey (eg, for the 2005 survey, all men tested in 2004 and 2005 are included in the recent testers category). UAI, unprotected anal intercourse.

## Discussion

Rates of self-reported HIV testing increased among gay men in Scotland surveyed in commercial venues between 1996 and 2005, and correspond with the Scottish Government policy change to routine, opt-out testing in GUM clinics, which was introduced in the Scottish sexual health strategy.^5^ However, with increasing testing rates over time and a corresponding increase in recent testing, one might have expected to find higher proportions of those who have had a test, to have had one recently, but this was not the case. This, combined with the consistent trend that older age is associated with decreased likelihood of recent testing, is consistent with HIV testing being a one-off event. It is not indicative of HIV testing being a “routine” part of a sexual health check-up.

There are some limitations to consider when interpreting our results. This was a bar-based sample, so only men who visit the venues surveyed had the opportunity to participate and caution should be taken when generalising to the wider population of gay men. The analyses are also limited to the available variables included in all seven surveys; preventing the exploration of other potential confounding factors. Furthermore, our measure of recent testing would not necessarily pick up all those who had had their first test between studies, given that the gaps between the larger surveys are at least 2 years. However, our findings still have considerable implications for HIV testing and prevention policies.

**Key messages**

- HIV testing increased among gay men in Scotland between 1996 and 2005, from 49.7% to 57.8%, with recent testing increasing from 28.4% to 33.2%.
- The increase corresponds with the policy change to routine, opt-out testing in GUM clinics, but rates still compare unfavourably with near-universal levels elsewhere.
- Recent testing rates did not increase among ever testers and decreased with age, suggesting few men test repeatedly or regularly.
- Further efforts to increase regular HIV testing among gay men are essential, and must also challenge HIV stigma and “normalise” testing at the community level.

UK guidelines recommend routine, opt-out HIV testing in GUM settings,^7^ ^17^ and the offer of HIV testing to MSM in UK clinics increased by 35% between 2003 and 2006, with uptake reported to be 85%.^2^ However, at the community level in Scotland these changes have not yet achieved the near-universal testing seen in the USA and Australia.^12^ ^13^ It is possible that the increase in uptake in GUM clinics reflects more frequent testing in these settings rather than an increase in the absolute numbers of MSM being tested. This is appropriate given that in the community-based surveys of HIV prevalence the majority of men with undiagnosed HIV had previously tested negative, whereas levels of HIV were low among men who have never tested.^18^ Specialist sexual health services are ideally placed to target and recall men who test negative, but report high-risk sexual behaviour, for repeat testing. However, community, or scene-based, testing initiatives should also be trialled to increase testing uptake among men not accessing mainstream services.^19^ ^20^

HIV testing levels among gay men in Scotland could also be limited by factors specific to the Scottish context. In-depth research on the HIV testing behaviour of gay men in Scotland has demonstrated that fear of a positive result, along with HIV-related stigma and discrimination within the gay community, discouraged testing.^14^ ^21^ ^22^ In fact, within this particular cultural context the anticipated burden of coping with a positive diagnosis was understood to be more important in shaping testing decisions than accessing appropriate treatment and care.^23^

Our findings suggest that few men become regular or repeat testers. As would be expected, older men are more likely to have tested within their lifespan, but they are less likely than their younger counterparts to report recent testing. This may indicate that men are making informed decisions about the need to test based on their perceived risk or that there are age-related patterns in undiagnosed infection. Recent testing rates were higher among men who reported high-risk sexual behaviour, but even here under half of men (and only one-third of men aged 35–44 years) who reported UAI with multiple partners in the previous year had had a recent test. The limitations of serosorting (only having UAI with partners of the same status) as a risk-reduction strategy for HIV-negative and untested men have been widely reported,^18^ ^24^ ^25^ ^26^ ^27^ and men who have never, or not recently, tested cannot claim to know their status accurately if, as described here, they also report sexual risk behaviour. Receptive UAI, particularly with partners of unknown HIV status, is one of the main sexual behaviours identified as a risk factor for HIV seroconversion,^28^ ^29^ and therefore accurate knowledge of HIV status is essential if men are to avoid unknowingly putting others at risk of HIV.

Knowledge of HIV status is particularly important when levels of undiagnosed HIV infection are high. Recent data (measured through oral fluid samples collected and anonymously tested for HIV antibodies) show 48.1% of HIV-positive men in bar-based surveys in Glasgow, and 36.4% in Edinburgh, are undiagnosed.^18^ The proportion undiagnosed decreases with age, but with higher overall prevalence in older men, the prevalence of undiagnosed HIV is twice as high in older than younger men in community-based surveys (average of 4.5% in the 26–35 and 1.9% in the 15–25 years age group).^18^ Given that among UK MSM, HIV diagnoses remains highest in the 25–34 and 35–44 years age groups,^2^ there is a clear need to promote regular HIV testing further.

Testing and treatment decisions are complex,^30^ but early diagnosis is essential to ensure timely access to treatment. In 2006, 20% of MSM diagnosed with HIV had a CD4 cell count less than 200 cells/mm^3^ (which at the time was the recommended threshold for treatment to commence), and men diagnosed late are 14 times more likely to die within a year of diagnosis than those diagnosed earlier.^2^ Nevertheless, efforts to increase regular HIV testing have to promote the positive benefits of knowing your HIV status beyond simply having access to treatment.

Accurate knowledge of status allows men to make effective use of primary and secondary prevention strategies, such as serosorting and postexposure prophylaxis. It should also allow them to make informed sexual risk decisions and be better able to negotiate sex through effective status disclosure, to avoid unknowingly putting others at risk of HIV. However, we have previously demonstrated that gay men in Scotland exhibit undue confidence that their partners share the same assumed negative status as themselves,^15^ and, as previously noted in this paper, there are continuing issues around HIV stigma and discrimination within the gay community.^14^ ^21^ These remain significant barriers to efforts to increase HIV testing further among gay men in Scotland.

Increasing testing may not be enough in itself to reduce HIV infections among UK gay men, when sexual risk is reportedly higher among men living with diagnosed HIV,^18^ but the promotion of regular HIV testing should be an essential component of HIV prevention, and additional, innovative efforts are required to increase its uptake. These efforts must do as much to challenge HIV stigma and “normalise” regular testing at the community level as they do to increase uptake at the individual level.
